# Death of Dr. Osborn

**Published:** 1902-09

**Authors:** 


### DEATH OF DR. OSBORN.

The venerable and beloved Dr. T. C. Osborn died at his home in Cleburne, Texas, August 19th ult., aged 85. He was one of the patriarchs of the profession, and one of the veterans of the medical corps of the Confederate army. He had long been an honorary member of the Texas State Medical Association. It is a satisfaction to his friends to know that after a long life of labor and great usefulness he passed away without a pang or a struggle, as peacefully as an infant falling asleep on its mother's breast. Dr. Osborn left a monument to his memory more enduring than brass,—he has a niche in the Temple of Fame side by side with Jenner; for he discovered the proper,—the only rational treatment for smallpox, both its prevention and cure—its abortion. His name will go sounding down the ages as man's benefactor in that he taught the present and all coming ages how to rob the red pest of its terrors.

He was one of the old-time gentlemen. He represented in his manner, his character, his intercourse with his fellowmen the best element of the illustrious Old South. He belonged in the category with the great, the beloved, Lee, whom he much resembled in appearance. I never saw him but that I thought it was a mistake that he had not on a ruffled shirt and knee breeches and pumps with big silver buckles. They were the fashion in his youth and he doubtless wore them.

Eighty-five years! And sixty years of the practice of medicine! How reminiscent of "calomel and jalap" and the lancet; of "salts and senna," of "clysters" and "poultices." Dr. Osborn was famous when I was a boy. The first time I ever heard of him was when he was a young man practicing in Greensboro, Alabama. He had a case of "malarial earache" in a child, and he had exhausted the resources of his saddle bags without relief. He took out his tobacco to take a chew and, think, when the idea occurred to him that tobacco is a powerful narcotic sedative. He sent the mother from the room on some pretext, and, bending over the little sufferer, instilled into its aching ear a warm *salivary decoction of tobacco* from his mouth. The relief was immediate. Later he made a decoction (without masticating, however), and it became famous throughout Alabama as "Dr. Osborn's Earache Remedy."

The following I take from *The Cleburne Morning Review,* August 20, 1902:

Tuesday night at 11:45 o'clock Dr. T. C. Osborn passed peacefully away at his home at 714 North Robinson street. It was the closing of a most eventful life, the stilling of hands that have done noble work for the medical profession of Texas and of the whole country. He was the discoverer of the bichloride treatment for smallpox which will give him a name in history. He was also the author of the "Malarial Tongue," and many other essays and treatments of disease. He wrote a great deal and had been a practitioner for fifty years.

He was born in Rutherford county, Tennessee, in 1818, and moved to Greensboro, Ala., where he practiced medicine for many years. All of his children were born in Alabama. He moved to Texas in 1882 and has resided here ever since. He had a very large circle of friends and the announcement of his death will cause much sorrow. His death was due to heart failure and nervous prostration. There was not a struggle; death came as an angel of peace and bore his spirit to its happy reward.

Deceased leaves the following children: Dr. J. D. Osborn, T. H. Osborn and Miss Ethel Osborn, of this city; Mrs. Laura Mason, of Fort Worth, and Mrs. J. S. Taylor, of Dallas.

